# Evaluation of alpha-II-spectrin breakdown products as potential biomarkers for early recognition and severity of aneurysmal subarachnoid hemorrhage

**DOI:** 10.1038/s41598-018-31631-y

**Published:** 2018-09-06

**Authors:** Linda Papa, Kimberly Rosenthal, Francesca Silvestri, John C. Axley, Jared M. Kelly, Stephen B. Lewis

**Affiliations:** 1 Department of Emergency Medicine, Orlando Regional Medical Center, Orlando, Florida United States; 2 Department of Neurology and Neurosurgery, McGill University, Montreal, Quebec Canada; 3 Perth Neurosurgery, Nedlands, Western Australia Australia; 4 Department Neurosurgery, University of Florida, Gainesville, Florida United States

## Abstract

This study assessed whether cytoskeletal protein alpha-II spectrin breakdown products (SBDP150, SBDP145, and SBDP120) would identify the presence of aSAH and be associated with severity (GCS score, WFNS grade and survival to hospital discharge). This prospective case-control study, conducted at a tertiary care Level I trauma center, enrolled adult patients with angiography confirmed aSAH who underwent ventriculostomy placement for cerebrospinal fluid (CSF) drainage. There were 40 patients enrolled in the study, 20 with aSAH and 20 control subjects. Patients with aSAH were a mean age of 54 (SD15) and 75% were female. There were significant differences in SBDP150, SBDP145, and SBDP120 CSF levels between patients with and without aSAH (p < 0.001), even in those presenting with a GCS Score of 15 and a WFNS Grade 1. The AUC for distinguishing aSAH from control subjects was 1.0 for SBDP150 and SBDP145, and 0.95 for SBDP120. SBDP150 and SBDP145 both yielded sensitivities and specificities of 100% and SBDP120 was 90% and 100% respectively. Moreover, there were significantly higher levels of SBDP150 and SBDP145 in the non-survivors than in the survivors (p < 0.001). This study demonstrates the potential that SBDP’s have as biomarkers for recognition and severity of aSAH. A larger prospective study is warranted.

## Introduction

The worldwide incidence of aneurysmal subarachnoid hemorrhage (aSAH) is approximately 9 per 100,000 persons/year, and the incidence of new cases in the United States is about 30,000 persons/year or 10/100,000 persons/year^1,2^. It is estimated that 80% of aSAH cases result from ruptured aneurysms and delays in treatment can increase the risks of rebleeding, disability and death^3,4^. Therefore, early detection is critical to reducing morbidity and mortality from this condition.

Approximately 1% to 6% of all patients who present to an emergency department with a headache will have an aSAH^5,6^. It is not uncommon for aSAH to be initially mistaken for acute migraine^7^ and the recognition of aSAH in patients with an otherwise normal neurological examination can be particularly difficult. Rates of misdiagnosis lay between 5–12% but can be as high as 51%^7^. Commonly used diagnostic tools in the emergency department include lumbar puncture, CT scanning, and angiography^6^. There is currently no biomarker available to aid emergency physicians in the detection of aSAH^8^. Given the dire consequences of missed diagnoses of aSAH, the development of an objective test which is non-invasive, simple and reliable is could improve the timely delivery of appropriate treatment.

During periods of necrosis or apoptosis, as seen in aSAH and other intracranial pathologies, there is activation of caspases and calpains which begin cleaving many cellular proteins including alpha-II- spectrin^9,10^. One of the many proteins that calpain-2 and caspase-3 are known to cleave is the cytoskeletal αII-spectrin protein^11,12^. Alpha-II-spectrin (280 kDa) is a cytoskeletal component of the cortical membrane of presynaptic terminals and axons. The proteolytic dissolution of this neuronal protein produces a 150KDa, 145 KDa and 120 KDa breakdown product (SBDP150, SBDP145, and SBDP120) that have previously been studied as markers of injury severity after TBI and have been found to correlate with injury severity in severe TBI patients as determined by CT infarct volume, GCS scores, and 6-month outcome^13–17^. It has also been shown that SBDP150 serum levels in patients after a mild TBI are related to short term measures of injury severity such as Glasgow Coma Scale (GCS) score, presence of intracranial injuries as reported by CT, and neurosurgical intervention^18^.

These biomarkers have also been examined in patients with aSAH relative to development of vasospasm^8,19,20^. SBDP concentrations increased from baseline levels for up to 12 hours prior to the clinical detection of cerebral vasospasm, and decreased to baseline levels after successful treatment^19^. This suggests a role in the early diagnosis of cerebral vasospasm-induced cerebral ischemia.

This study aimed to assess the diagnostic and prognostic utility of SBDPs as potential biomarkers for aSAH by examining: (1) the relationship between early CSF levels of SBDP in patients with aSAH compared to controls, (2) differences between patients with different levels of initial aSAH severity (GCS score and WFNS grade), and (3) survival to hospital discharge.

## Methods

This prospective case-control study was conducted at a tertiary care Level I Trauma Center over a 12-month period. Adult patients presenting to the emergency department with confirmed aSAH requiring an external ventricular drain were eligible for enrollment. The diagnosis of aSAH was based on results from CT angiography and/or cerebral angiography. Patients were enrolled within 72 hours of the estimated time of aneurysm rupture (headache), and the first sample was obtained at the earliest available sample time-point from enrollment. Additional samples were obtained every 6 hours over the course of 14 days. Control subjects were age-matched patients undergoing spinal anesthesia for peripheral vascular procedures and had CSF samples obtained as part of their routine care. The study was approved by the University of Florida institutional review board and the study was performed in accordance with ethical standards and federal regulations for human research. Informed consent was obtained from all patients or their legally authorized representatives prior to enrollment.

### Biomarker Analysis

CSF samples were taken from the initial placement of the drain then stored on ice for up to 12 hours before being centrifuged and frozen at −80C. Samples were coded using a bar code cataloguing system to assure patient confidentiality and transferred for analysis to a central laboratory. SBDP’s were measured using Western Blot technique and quantified in arbitrary densitometric units (ADU’s) and has been previously described^19^. Samples were prepared and resolved using sodium dodecylsulfate polyacrylamide gel electrophoresis. After electrophoresis, separated proteins were laterally transferred to polyvinylidene fluoride membranes which were probed using an anti–aII spectrin monoclonal antibody (alpha-fodrin, Biomol International) that detects intact nonerythroid aII-spectrin (280 kD) and 150-, 145-, and 120-kD (SBDP150, SBDP145, and SBDP120, respectively) fragments. Enhanced chemiluminescence reagents (obtained from Amersham Biosciences) were used to visualize immunolabeling on Biomax ML chemiluminescent film (Kodak). Semiquantitative evaluation of protein levels detected on immunoblotting was performed using computer-assisted densitometric scanning (Expression 1640XL, Epson). Data were acquired as integrated densitometric values using commercially available computer software (ImageJ, version 1.60; National Institutes of Health) and transformed into percentages of the mean densitometric values obtained from control CSF samples. Therefore, control samples from the CSF were tested alongside those obtained from the study participants for direct comparison on immunoblotting.

### Outcome Measures

The primary outcome measure was the presence of acute aSAH on CT angiography and/or cerebral angiography compared to control subjects without aSAH. Board certified neuroradiologists, who were unaware of the study, interpreted the CT’s as per usual practice. The secondary outcome measure was clinical severity of the aSAH based on the WFNS (World Federation of Neurosurgical Societies) grading system which uses the Glasgow Coma Scale and presence of focal neurological deficits to grade the severity of subarachnoid hemorrhage^21^. Grade 1 is a GCS 15 without deficit, Grade 2 is a GCS 13–14 without deficit, Grade 3 is a GCS 13–14 with focal neurological deficit, Grade 4 is a GCS 7–12, with or without deficit, and Grade 5: GCS <7 is with or without deficit. Survival to hospital discharge was also evaluated.

### Statistical Evaluation

Descriptive statistics including means and medians, together with 95% confidence intervals and inter-quartiles ranges [IQR] were used to describe the data. Clinical and biomarker data were assessed for distribution and variance. Univariate analysis was conducted using Mann Whitney U and Kruskal-Wallis Test. Receiver Operating Characteristics (ROC) Curves were constructed to determine how well the biomarkers discriminated between the outcome measures, including presence of subarachnoid hemorrhage, WFNS grade, and survival to hospital discharge. Performance was also assessed by sensitivity, specificity, positive and negative predictive values with 95% confidence intervals. Significance was set at 0.05.

Post hoc group sample size calculations of 20 (aSAH) and 20 (controls) achieved a 100% power (beta) to detect a difference of 64.2 ADU between the null hypothesis and the alternative hypothesis with known group standard deviations of 0.9 and 37.3 and with a significance level (alpha) of 0.05.

## Results

A total of 40 patients were enrolled in the study, 20 with aSAH and 20 without aSAH. Patients were a mean age of 54 (SD15) and 75% were female. Four patients with aSAH (20%) presented to the emergency department with a GCS score of 15 and a WFNS Grade I. A description of the of these aSAH patients is shown in Table 1. There were no significant differences in the characteristics between those presenting with a GCS 15 (WFNS Grade I) and those with GCS 3–14 (WFNS Grade II–V). Initial CT scans confirmed significant amounts of subarachnoid blood products (clots more than 1-mm thick) in combination with intraventricular hemorrhage, consistent with Fisher Grade 3 in all study patients.

**Table 1:** Characteristics of aneurysmal SAH patients enrolled in the study.

	Initial GCS Score 15 (WFNS Grade I) N = 4	Initial GCS Score 3–14 (WFNS Grade II–V) N = 16	Total N = 20	P-Value
Age	57 (SD17)	54 (SD15)	54 (SD15)	0.683
	[Range 34–76]	[Range 33–78]	[Range 33–78]	
Gender (% female)	4 (100%)	11 (69%)	15 (75%)	0.530
Treated with				
Clip	3 (75%)	10 (63%)	13 (65%)	0.832
Coil	1 (25%)	5 (31%)	6 (30%)	
None	0 (0%)	1 (6%)	1 (5%)	
Survived to Hospital Discharge	1 (25%)	10 (63%)	11 (55%)	0.285

Demographic and clinical data for all subjects included in the study.

**Figure 1:**
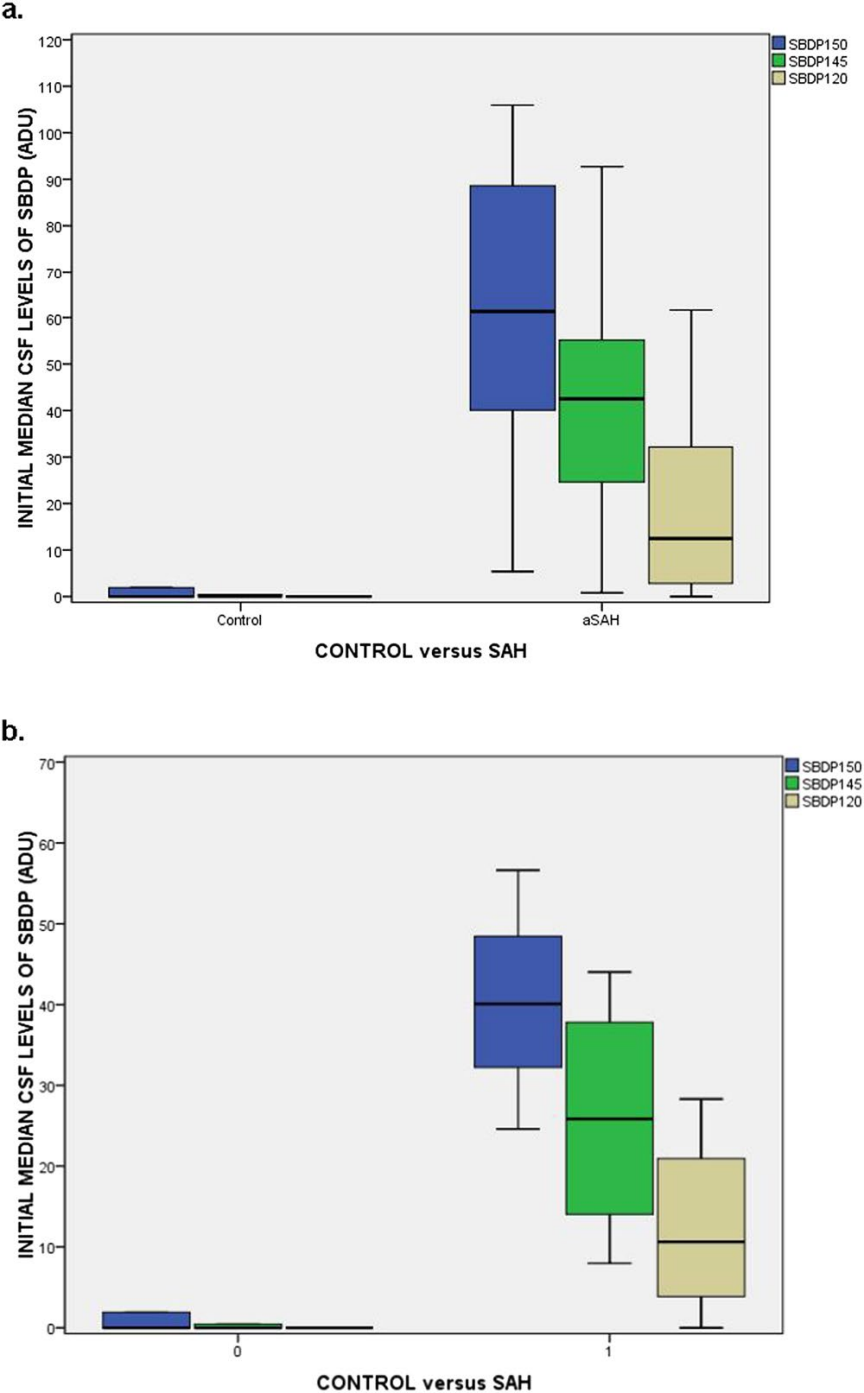
(**a**) Boxplot of initial CSF levels of SBDP150, 145 and 120 in control patients versus aSAH patients. Patients with aSAH had significantly higher initial CSF levels of all three SBDP’s (p < 0.001) compared to control patients. Boxplots represent medians with interquartile ranges. (**b)** Boxplot of initial CSF levels of SBDP150, 145 and 120 in control patients versus aSAH patients presenting with a GCS 15. Patients with aSAH who presented with a GCS 15 and WFNS Grade I had significantly higher initial CSF levels of all three SBDP’s (p < 0.001) compared to control patients. Overall, CSF levels of SBDP150, SBDP145, and SBDP120 were lower in those presenting with GCS 15 compared to all GCS scores. Boxplots represent medians with interquartile ranges.

**Figure 2:**
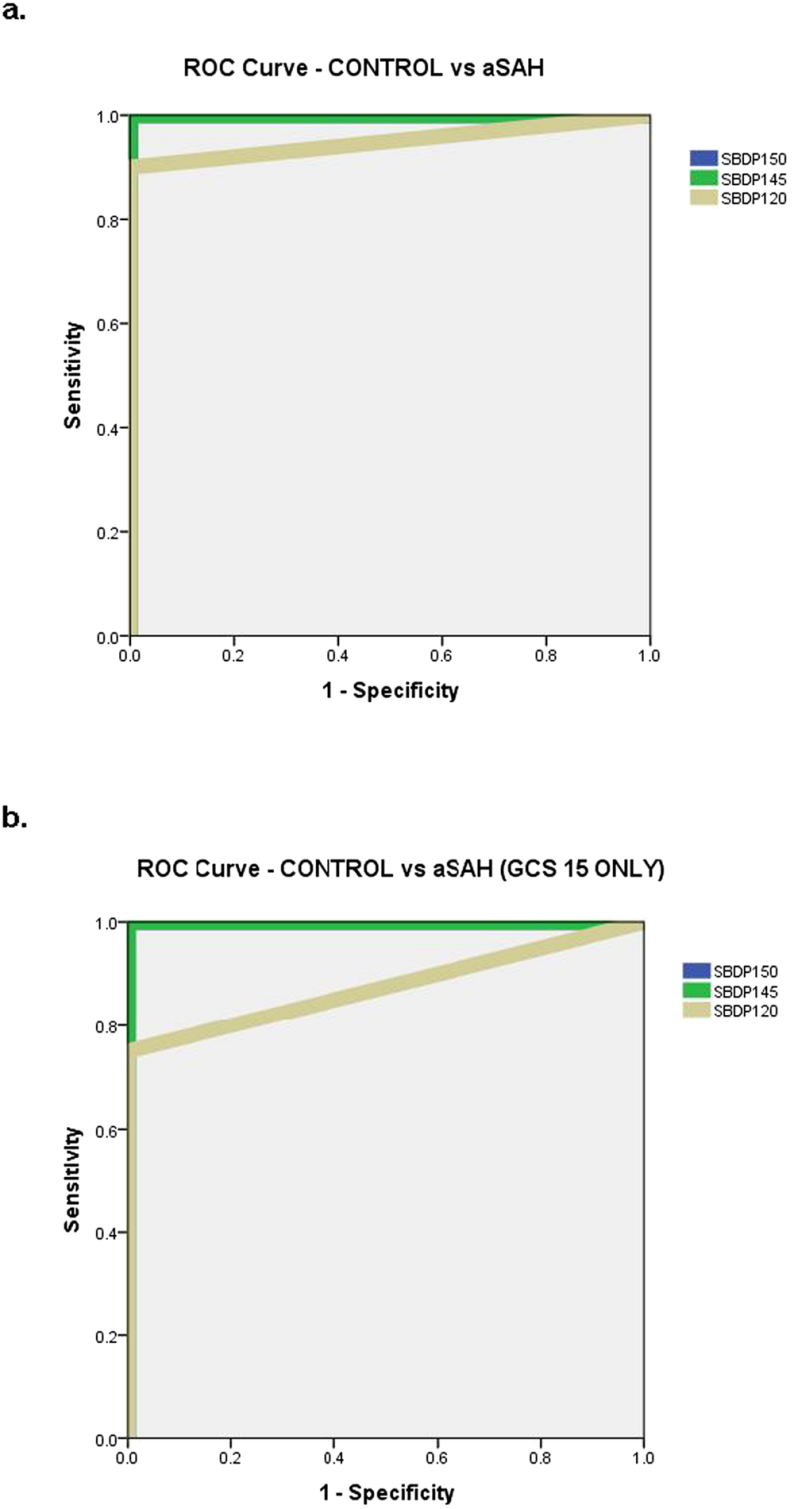
(**a**) ROC Curve for distinguishing control patients from aSAH. The Area under the ROC Curve (AUC) for SBDP150 was 1.0 (95% CI = 1.0–1.0), for SBDP145 1.0 (95% CI = 1.0–1.0), and for SBDP120 0.95 (95% CI = 0.87–1.0). (**b**) ROC Curve for distinguishing control patients from aSAH in those presenting to the ED with a GCS 15. The Area under the ROC Curve (AUC) for SBDP150 was 1.0 (95% CI = 1.0–1.0), for SBDP145 it was 1.0 (95% CI = 1.0–1.0), and for SBDP120 it was 0.88 (95% CI = 0.62–1.0).

There were significant difference in SBDP150, SBDP145, and SBDP120 CSF levels between patients with and without aSAH (p < 0.001) (Fig. 1a). Moreover, in those aSAH patients presenting with a normal mental status (GCS Score of 15) and no neurologic deficits (WFNS Grade I) levels of SBDP150, SBDP145, and SBDP120 were still significantly elevated compared to controls (p < 0.001) (Fig. 1b). The area under the ROC Curve was calculated to evaluate the ability to distinguish aSAH from control subjects and was found to be 1.0 (95% CI = 1.0–1.0) for SBDP150, 1.0 (95% CI = 1.0–1.0) for SBDP145, and 0.95 (95% CI = 0.87–1.0) for SBDP120 (Fig. 2a). In those patients presenting with GCS 15, AUC’s for SBDP150 and SBDP145 remained unchanged and for SBDP120 the AUC was 0.88 (95% CI 0.62–1.0) (Fig. 2b).

**Figure 3:**
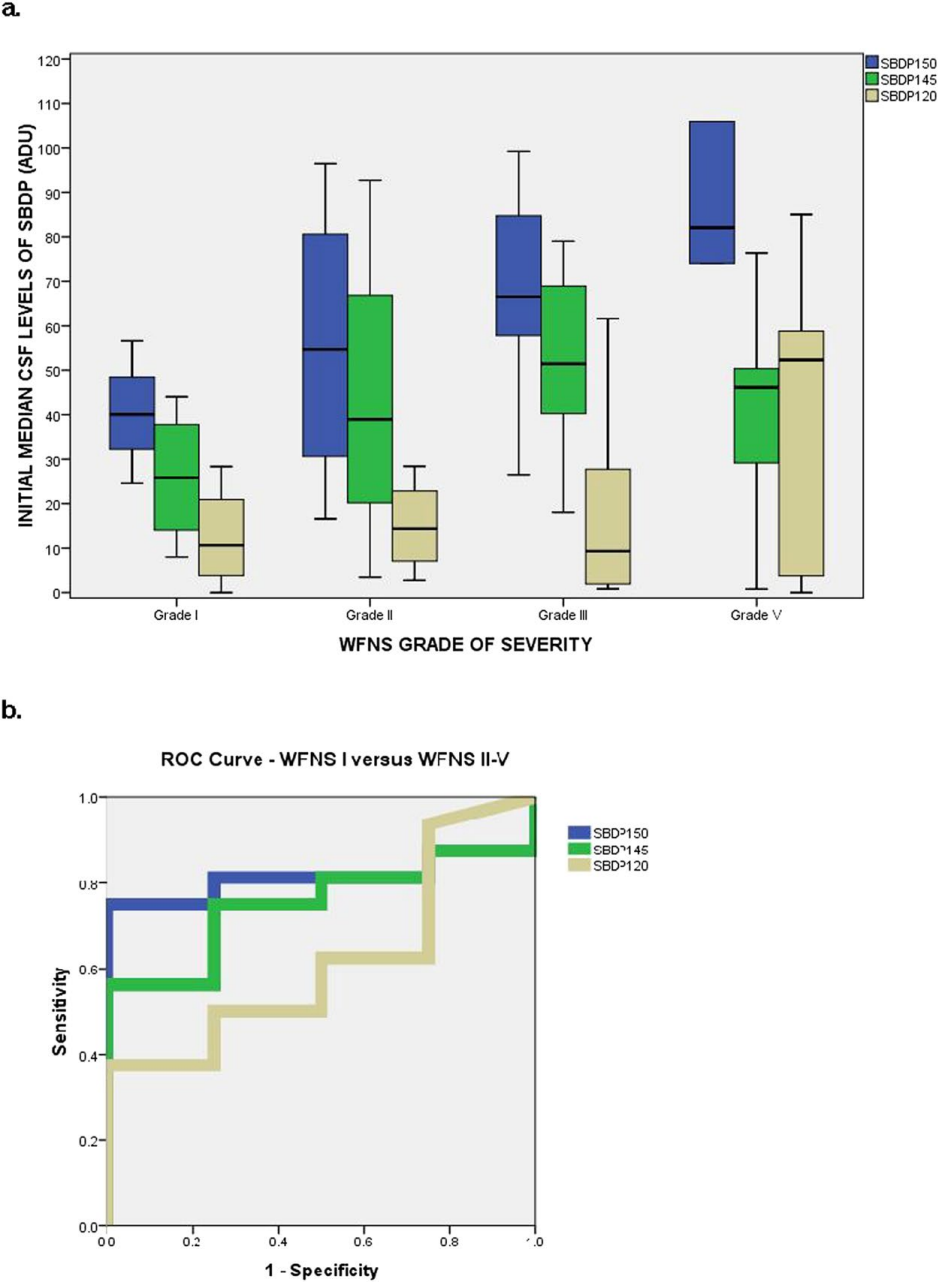
(**a**) Boxplot comparing initial levels of SBDP150, SBDP145, and SBDP120 in different WFNS grades of aSAH severity at initial presentation to the ED. Levels of SBDP150, SBDP145, and SBDP120 were highest among patients with WFNS grade IV and V compared to those with Grade I or II but the differences were not statistically significant p = 0.057, p = 0.181 and p = 0.571 respectively. WFNS Grade 1 is a GCS 15 without deficit (n = 4), Grade 2 is a GCS 13–14 without deficit (n = 4), Grade 3 is a GCS 13–14 with focal neurological deficit (n = 0), Grade 4 is a GCS 7–12, with or without deficit (n = 7), and Grade 5: GCS < 7 is with or without deficit (n = 5). Boxplots represent medians with interquartile ranges. (**b**) ROC Curve for distinguishing patients with WFSN Grade I versus patients with WFNS Grades II-V. The AUC for differentiating WFNS Grade I from Grade II-V was 0.81 (95% CI 0.63–1.0) for SBDP150, 0.75 (95% CI 0.54–0.0.97) for SBDP145, and 0.62 (95% CI 0.34–0.90) for SBDP120.

Initial levels of SBDP150, SBDP145, and SBDP120 were compared by different WFNS grades of aSAH severity at initial presentation to the emergency department. Levels of SBDP150, SBDP145, and SBDP120 are associated with increasing levels of WFNS grades of severity (Fig. 3). Levels of SBDP150, SBDP145, and SBDP120 were highest among patients with more severe WFNS grades IV and V compared to those with Grade I or II but the differences were not statistically significant (p = 0.057, p = 0.181, and p = 0.571 respectively). The AUC for differentiating WFNS Grade I from Grade II-V was 0.81 (95% CI 0.63–1.0) for SBDP150, 0.75 (95% CI 0.54–0.0.97) for SBDP145, and 0.62 (95% CI 0.34–0.90) for SBDP120 (Fig. 3b).

Cutoff points for SBDP’s were derived from the ROC Curves for detecting distinguishing aSAH from control patients to maximize the sensitivity. At a cutoff level of 2.0 ADU, SBDP150 yielded a sensitivity of 100% and a specificity of 100% (Table 2). At a cutoff level of 0.5 ADU, SBDP145 also yielded a sensitivity of 100% and a specificity of 100% (Table 2). At a cutoff level of 0.5 ADU, SBDP120 yielded a sensitivity of 90% and a specificity of 100% (Table 2).

**Table 2:** Contingency tables for CSF SBPB150, SBDP145, and SBDP120 in detecting aneurysmal SAH from control patients without aSAH.

SBDP150	aSAH	No aSAH
SBDP150 positive >2.0	20	0
SBDP150 negative ≤2.0	0	20
Sensitivity	100% (80–100)	
Specificity	100% (80–100)	
**SBDP145**	**aSAH**	**No aSAH**
SBDP145 positive >0.5	20	0
SBDP150 negative ≤0.5	0	20
Sensitivity	100% (80–100)	
Specificity	100% (80–100)	
**SBDP120**	**aSAH**	**No aSAH**
SBDP145 positive >0.5	20	0
SBDP150 negative ≤0.5	2	18
Sensitivity	90% (67–98)	
Specificity	100% (80–100)	

Contingency tables for CSF SBPB150, SBDP145, and SBDP120 in detecting aneurysmal SAH from control patients without aSAH.

**Figure 4:**
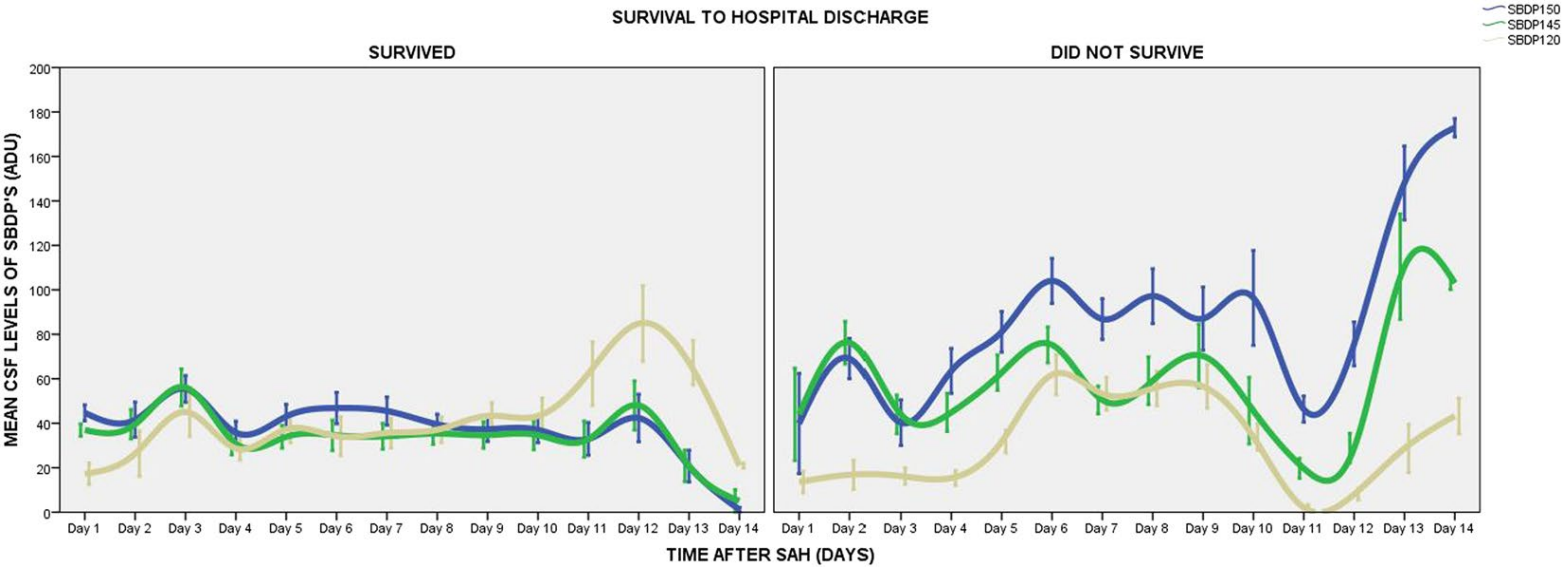
Temporal profile of SBDP150, SBDP145, and SBDP120 is compared in survivors versus non-survivors at hospital discharge. There are significantly higher levels of SBDP150 and SBDP145 in the non-survivors than in the survivors (p < 0.001) over 14 days but levels were not significantly different for SBDP120 (p = 0.830). Lines represent means with standard deviations.

The prognostic ability of SBDP150, SBDP145, and SBDP120 to predict survival to hospital discharge was assessed by comparing their temporal profiles over 14 days (Fig. 4). There were significantly higher levels of SBDP150 and SBDP145 in the non-survivors than in the survivors (p < 0.001) over 14 days but levels were not significantly different for SBDP120 (p = 0.830). Maximal levels in survivors were 166, 180, and 192 ADU for SBDP150, SBDP145, and SBDP120 respectively and in non-survivors maximal levels were 225, 224, and 212 ADU respectively. There were initial elevations in the first 3 days of rupture in both survivors and non-survivors but the levels were highest in non-survivors. Survivors demonstrated steady elevations with mean levels between 20 and 60 ADU from Day 4 to Day 10. In contrast, non-survivors had significant elevations with multiple peaks and troughs from Day 4 to Day 10 with mean levels between 60 and 120 ADU. The levels of the 3 markers showed marked decreases after Day 12 in those who survived and marked elevations in those who did not survive to discharge. The AUC for initial levels of SBDP’s predicting survival to hospital discharge was 0.64 (95% CI = 0.38–0.89) for SBDP150, 0.71 (95% CI = 0.47–0.95) for SBDP145, and 0.36 (95% CI = 0.10–0.61) for SBDP120.

## Discussion

This study assessed the ability of breakdown products of the neuronal cytoskeletal protein alpha-II spectrin (SBDP’s) to distinguish between patients with and without aSAH in a population of patients who presented to the emergency department and were diagnosed with aSAH. One of the primary objectives of this study was to establish whether early SBDP150, SBDP145, and SBDP120 levels would be elevated in those who presented with an otherwise normal mental status as operationally defined by a GCS of 15 and no neurologic deficits (WFNS Grade I). It was important to evaluate these markers as early after rupture as possible, as this is the period when they could be most useful in identifying rupture and also predict severity of hemorrhage. A subgroup of separately analyzed patients, who presented with a GCS score of 15 and WFNS Grade 1, was indeed found to possess significantly higher amounts of CSF SBDP’s than control subjects (p < 0.001). SBDP150 yielded a sensitivity of 100% and a specificity of 100%, SBDP145 also yielded a sensitivity of 100% and a specificity of 100% and SBDP120 yielded a sensitivity of 88% and a specificity of 100%.

Despite the limited receptivity of the GCS to subtler cues of neurological injury, it is still a common measure of mental status is the ED^22^. A GCS score of 15 can create a false sense of “normality” and puts these patients at risk of undergoing less comprehensive and delayed investigations than those who present with a score below 15 thus increasing the risk of brain damage from delayed care. The diagnostic challenge for emergency physicians thus lies in patients whose presentation of aSAH is obscured by normal neurological examinations and acute headache seemingly symptomatic of migraine^22^.

Rapid appearance of a biomarker in biological material is imperative which why we elected to examine the initial or “earliest” time-point available for analysis. This approach has been used in previous TBI studies^17,23^. Of note, initial levels of SBDP150, SBDP145, and SBDP120 were associated with increasing levels of initial WFNS grades, indicating that concentrations of the biomarkers were associated with the severity of the aSAH. Although, we did not obtain samples within 4 hours of rupture in this study, a previous clinical study demonstrated that SBDP150 was detectable in serum of mild TBI patients within 4 hours of injury and therefore has the potential to be used as an early marker^18^. Future studies should obtain samples earlier.

SBDP150, SBDP145 and SBDP120 have shown promise as potential markers for TBI as their concentrations in the CSF have been strongly correlated with clinical outcomes and with injury severity^13–17^. This is consistent with the findings of this study as applied to aSAH. SBDP150 and SBDP145 measured within 24 hours of severe TBI have been shown to significantly predict 3–6 month mortality with an AUC of 0.68 and 0.74 respectively^16,17^. Accordingly, our study has shown that SBP150 and SBDP145 predicts mortality to hospital discharge with an AUC of 0.64 and 0.71 respectively. There are, however, differences in the behavior of apoptotic marker SBDP120 with AUC of 0.61 in TBI and 0.36 in aSAH^17^. The observed differences in the time frame at which calpain (a necrosis marker yielding SBDP150/145) and the caspase-3 (an apoptosis marker yielding SBDP120) become activated are apparent in the temporal profile of TBI and aSAH patients. In the aSAH patients initial peaks of the three biomarkers were observed within the first 3 days of rupture in both survivors and non-survivors but the levels were highest among non-survivors. The subsequent pattern of release after Day 4 contrasted significantly between survivors and non-survivors. It is interesting that the pattern of biomarker release of SBDP150 and SBDP145 are quite similar. Although SBDP120 does resemble the expression of SBDP150 and SBDP145, levels are much lower and tend to peak later. This possibly reflects calpain activity (SBDP150 and SBDP145) versus caspase-3 activity (SBDP120). Similar patterns can be seen in patients with survival form severe TBI with biomarker release patterns reflecting the severity of the initial insult and secondary insults^15,17^. These findings suggest that, in patients with aSAH, the temporal profile of these biomarker changes is an important predictor of clinical outcome. Survival indicators could serve to help clinicians prognosticate and could potentially be used to gauge therapy.

Two biomarkers (GFAP and UCH-L1) that received FDA approval in early 2018 to predict CT lesions in mild to moderate TBI within 12 hours of injury also appear to have potential implications for use in stroke^24–27^. Numerous findings have suggested that the more immediate structural damage to the artery and blood-brain barrier caused by hemorrhage in comparison with ischemic stroke renders the subsequent surge in GFAP levels a reliable indicator of hemorrhage specifically^24,28–31^. Lewis *et al*. found that individuals suffering from aneurysmal subarachnoid hemorrhage (aSAH) had consistently higher concentrations of Ubiquitin C-terminal Hydrolase (UCH-L1) in the CSF two weeks after post-aneurysmal rupture, which were significantly associated with poor recovery^32^. Siman *et al*. similarly found that CSF concentrations of UCH-L1, taken over a 10-day period since aneurysmal rupture, rose significantly and predicted severity of infarction, vasospasm, and outcome^20^.

The use of biomarker panels in the detection of aSAH offers a further advantage in its joint accuracy and precision, a combination not often observed through the use of scales and head rules alone. In a 2017 study, Perry *et al*. explored the utility of their recently validated Ottawa SAH Rule, similarly aspiring towards a diagnostic system which both moved away from dependence on neuroimaging and captured otherwise neurotypical SAH patients presenting with acute headache. While the Ottawa SAH Rule was found to offer 100% sensitivity, its specificity of 13.6–15.9% demonstrates the need and opportunity for highly sensitive and specific biomarker testing panels to be employed as diagnostic tools alongside ever more accurate injury scales, particularly when both systems share similar procedural goals for the same at-risk target group. Further studies are thus warranted which explore the success of a joint clinical guideline and biomarker panel diagnostic system. Moreover, the implementation of biomarker testing panels conveys an additional opportunity in its potential prognostic use.

While these data are encouraging, the authors recognize there are limitations to this study. Perhaps the most profound drawback to employing CSF biomarkers is that CSF samples are not as easily or quickly acquired as blood. CSF samples obtained through lumbar puncture are invasive, time-consuming and typically uncomfortable. SBDP150 and SBDP145 are detectable in serum following TBI and future studies should use employ blood samples^16,17^.

This study is further complicated by the fact that control patients did not present with headache, and considering the strong potential of biomarkers in migraine detection, it is especially important to continue to tease out the performance differences between various biomarkers for aSAH and acute-onset migraine. It must also be noted that potential confounds might have arisen from an inability to completely match subjects and controls due to the unavailability of demographic information for control subjects and the fact that a majority of aSAH patients were female. Further work must be undertaken to develop biomarker testing assays and panels so that values such as positive likelihood ratio can be measured for biomarker tests and thus facilitate a more sophisticated evaluation of the sensitivity and specificity of the biomarker as a diagnostic examination.

Although a post hoc power calculation showed that there was an adequate number of patients to satisfy the primary outcome, a prospective study employing a much larger sample size will be needed to make appropriate clinical correlations and reduce statistical noise.

## Conclusion

Early CSF levels of breakdown products of the neuronal cytoskeletal protein alpha-II spectrin (SBDP150, SBDP145, and SBDP120) showed potential in serving as indicators of aSAH, including in individuals presenting with normal mental status. Elevated SBDP levels were also associated severity of hemorrhage and early mortality. This is an important step towards earlier identification of aSAH which should be explored in the setting of a much larger prospective study.

## Data Availability

The dataset generated and analysed during the current study are not publicly available but are available from the corresponding author on reasonable request.
